# Quality of life in patients with progressive supranuclear palsy: a review of literature and implications for practice

**DOI:** 10.3389/fneur.2024.1476488

**Published:** 2024-11-20

**Authors:** Michał Markiewicz, Natalia Madetko-Alster, Piotr Alster

**Affiliations:** Department of Neurology, Medical University of Warsaw, Warsaw, Poland

**Keywords:** atypical parkinsonism, quality of life, progressive supranuclear palsy, neurodegenerative diseases, tauopathy, patient-reported outcomes

## Abstract

Progressive supranuclear palsy (PSP) is an atypical form of parkinsonism characterized by tauopathy, manifesting as oculomotor dysfunction, postural instability, akinesia, and cognitive/language impairments. The diagnosis and examination of PSP can be challenging, primarily due to the unclear and underexplored pathomechanisms involved, alongside absence of effective treatments. Clinical variants of PSP is the second most common form of neurodegenerative parkinsonism after Parkinson’s disease (PD). It is defined by a symmetrical akinetic-rigid syndrome (atypical parkinsonism) and vertical supranuclear gaze palsy. In contrast to PD, PSP often presents with gait instability, backward falls, and cognitive and behavioral changes at early disease stages. The classification of PSP has evolved since Richardson, Steele, and Olszewski’s initial reporting of the condition in 1963, which included a cohort of nine patients. Over the years, the definition of this disorder has evolved to encapsulate a group of patients with distinct clinical variants, notably the classical Richardson syndrome (RS) and several atypical phenotypes, each with significant implications for disease progression and quality of life (QoL). The 2017 Movement Disorder Society Diagnostic Criteria by Hoglinger et al., improved the sensitivity for detecting early and variant PSP presentations and provided more specific differential diagnoses for conditions such as PD and other forms of atypical parkinsonian syndromes. Owing to the growing interest in the disease’s progression, evaluating the QoL for patients with PSP has become crucial. This review emphasizes the significance of QoL evaluation and its feasibility for practical implications, serving as an initial foundation for future research focused on the well-being of individuals affected by PSP. Progressive supranuclear palsy (PSP) is an atypical form of parkinsonism characterized by tauopathy, manifesting as oculomotor dysfunction, postural instability, akinesia, and cognitive/language impairments. Diagnosing PSP is challenging owing to the lack of tools for differential examination. Additionally, the pathomechanism of this disease is not sufficiently understood, and no treatment is currently available. Owing to the growing interest in the disease’s progression, evaluating the quality of life (QoL) for patients with PSP has become crucial. This review emphasizes the significance of QoL evaluation and its feasibility for practical implications, serving as an initial foundation for future research focused on the well-being of individuals affected by PSP.

## 1 Introduction: clinical variants of PSP

Progressive supranuclear palsy (PSP) is a rare neurodegenerative parkinsonism with a prevalence of 1–18 per 100,000 compared to 1–2 per 1,000 in Parkinson’s disease (PD). It is characterized by symmetrical akinetic-rigid syndrome (atypical parkinsonism) and vertical supranuclear gaze palsy. In contrast to PD, PSP presents early with gait instability, backward falls, and cognitive and behavioral changes. Since 1963, when Richardson, Steele, and Olszewski (1) reported a series of nine patients, the definition of PSP has evolved to encompass a group of patients with distinct clinical variants. After the description of classical Richardson syndrome (RS), other specific atypical phenotypes of PSP have been discovered over the years (2). The recognition of different variants of PSP has expanded the concept into a group of several “subtypes,” with varied progression. These differences in the dynamics of patient deterioration significantly impact their quality of life (QoL).

The Movement Disorder Society Diagnostic Criteria, revised by Hoglinger et al. (4), has enhanced the sensitivity for detecting early and variant presentations of PSP, offering more precise differential diagnoses than the previous guidelines from the National Institute of Neurological Disorders and Stroke and the Society for PSP (NINDS-SPSP) (3). These guidelines emphasize four core functional domains: postural instability [P], oculomotor dysfunction [O], akinesia [A], and cognitive dysfunction [C]. By utilizing combinations of these domains, healthcare professionals can establish a diagnostic spectrum, classifying patients into three categories: probable PSP, possible PSP, and suggestive of PSP. Additional clinical indicators, such as levodopa resistance, dysphagia, spastic dysarthria, and photophobia, as well as specific radiological findings—such as predominant midbrain atrophy or hypometabolism and postsynaptic striatal dopaminergic degeneration—support the diagnostic process also play supportive roles in diagnosis. Among other symptoms, cognitive and behavioral abnormalities, including dysexecutive syndrome, bradyphrenia, impulsivity, disinhibition, perseveration, and reduced phonemic verbal fluency, can also complicate the assessment process. Currently, a neuropathological examination remains the only definitive diagnostic method for PSP (4).

Differentiating various PSP phenotypes is essential given the prognostic differences and implications for future drug trials. PSP–Richardson syndrome (PSP-RS) encompasses the characteristics originally described by Steele et al., where patients typically present with relatively early falls, cognitive dysfunction, gaze abnormalities, and postural instability (lurching and falls backwards) in the initial stages of the disease (5). PSP-RS is the most common variant, accounting for 76% of autopsy-confirmed cases in a recent series (6), and is associated with a poorer prognosis compared to the PSP-parkinsonism (PSP-P) phenotype, featuring an average disease duration of 5.9 years (ranging from 5 to 8 years) and an average age of death at 72.1 years (7). Conversely, PSP-P is characterized by primary asymmetrical tremor, non-axial dystonia, and early bradykinesia, with a notable response to levodopa therapy. These features resemble those of idiopathic PD. Patients with PSP-P tend to have a longer survival rate (9.1 years) compared to those with PSP-RS. However, the overlap in clinical characteristics between these two variants presents a significant diagnostic challenge. Neuropsychological assessments, such as those utilizing the Frontal Assessment Battery (FAB), can aid in identifying the two most common PSP variants (8). Moreover, due to insufficient data on clinical and pathological patterns, less common variants of PSP have not yet been formally detailed in the MDSPSP criteria (9); consequently, their low incidence means these variants are described only briefly in contemporary literature, typically in case reports or small case series. The most common variants of the disease are outlined in Table 1.

**Table 1:** Main symptoms found in the different PSP variants (1).

Richardson’s syndrome	PSP-parkinsonism (PSP-P)	PSP-corticobasal syndrome	PSP with predominant cerebellar ataxia (PSP-C)
Unsteady gait, falls, bradykinesia, impaired ocular movement (apraxia of eyelid opening, slowing of vertical saccades, vertical supranuclear gaze palsy) personality changes (apathy, disinhibition), cognitive slowing (bradyphenia), executive dysfunction (difficulty planning, multitasking), speech abnormalities (ataxic slow, spastic and hypophonic), dysphagia	Bradykinesia, tremor (asymmetric onset), rigidity, moderate initial response to levodopa treatment.	Asymmetric limb rigidity, alien limb, cortical sensory loss, dystonia, bradykinesia (unresponsive to levodopa), apraxia	Similar to MSA-C, but less pronounced autonomic dysfunction

PSP, progressive supranuclear palsy; MSA-C, multiple system atrophy-cerebellar type.

Despite significant progress in understanding PSP, achieving a definitive diagnosis still hinges on neuropathological examination. Achieving early and reliable diagnosis still remains a significant clinical challenge driven by both patient demands for ante-mortem confirmation and clinicians’ needs for effective therapeutic trials allocation. As a result, there is an urgent need for improved diagnostic tools. At present, no disease-modifying therapies are available for PSP, and only a limited number of treatments have proven effective. Levodopa can provide symptomatic relief, but its efficacy is typically limited and short-lived in PSP-P patients, with rare benefits seen in those with PSP-RS (10); the impact of this treatment on overall disease duration remains unclear. Physical therapy may yield measurable improvements on clinical rating scales (5). While apraxia of eyelid opening may be improved by pretarsal Botulinum toxin injections, these interventions do not address the major clinical symptoms that ultimately lead to fatality in all PSP cases.

PSP faces significant challenges in clinical examination due to the low specificity of *in vivo* diagnostic tools and the lack of evidence-based methods. There is growing interest in exploring other aspects of the disease, such as the quality of life, which is particularly relevant given the pronounced deterioration and incurable nature of PSP. This review aims to synthesize current knowledge regarding the methods of evaluation of QoL in PSP, using scales specifically dedicated for this clinical entity, as well as those generally applicable to parkinsonisms. Additionally, the significance of QoL assessment was examined in the context of its limitations and future perspectives to enhance understanding and intervention strategies.

## 2 Methods

We performed a search of the PubMed database for studies published from the date of inception of the database up to August 2024, searching for the following key words: “Progressive Supranuclear Palsy,” “Quality of Life,” and their abbreviations; and “movement disorders.”

## 3 Importance of QoL studies

Combining preclinical and clinical findings with QoL analysis enhances our understanding of the disease’s natural progression and a more precise identification of progression factors. Considered a vital adjunct to clinical data (11), QoL studies provide insights into neurodegenerative diseases beyond PD, which have been insufficiently documented largely due to the low prevalence of parkinsonian syndromes other than PD, and thus the unavailability of large cohorts (12). Gathering subjective data about patients’ well-being and other facets of their lives enables healthcare providers to deliver more effective and personalized clinical care. This approach has garnered endorsements from the Food and Drug Administration (FDA) and the World Health Organization (WHO) as vital for ongoing care improvement and future drug development (13). By prioritizing the patient’s perspective on disease progression, we can enhance management strategies. Given that many patients exhibit a lack of insight into their functional deficits (anosognosia)—a phenomenon well studied in extrapyramidal disorders, including PSP and Alzheimer’s disease (14)—numerical scales for assessing progression could prove valuable for accurately evaluating patient needs.

QoL is a broad, multidimensional concept that reflects patients’ subjective perceptions regarding how their disease affects their overall well-being. It encompasses physical, mental, emotional, and social dimensions. Self-reported tools for QoL assessment, which can be generic (applicable for numerous diseases, i.e., universal) or disease-specific (ie. disease oriented), are continually being developed for clinical studies and practice to quantify disease impact on patients’ everyday lives, treatment efficacy, and health policy management (15, 16). Some QoL aspects, such as symptom perception, self-image, and life satisfaction—including social and economic factors—are inherently subjective; however, others can be objectively measured, including clinical manifestations of the disease and patients’ financial and social situations. The instruments presented below include both subjective and objective indicators. While data on QoL in PSP is less comprehensive compared to sporadic PD (17), recent years have seen an uptick in interest surrounding QoL in Parkinsonism, and some tools for capturing QoL have been developed, mostly based on quantitative scales (18).

In addition to predominant motor symptoms, non-motor symptoms (NMSs) significantly increase morbidity and disability in PD and other parkinsonian disorders, considerably affecting the QoL for patients and their caregivers (19, 20). Researchers have observed variable patterns in the prevalence of specific NMS that differentially impact individuals’ lives. A study involving 50 patients with PSP compared to 100 patients with PD identified insomnia, fatigue, and urinary dysfunction as the most common NMSs in PSP (21). Other studies have also highlighted gastrointestinal problems and fatigue as prevalent issues (22). The authors of the largest PSP cohort study (23) found that the most prevalent domains in both the PSP-RS and PSP-P subgroups were sleep/fatigue and sexual function; however, mood/cognition was severely impaired in RS, while gastrointestinal issues were more pronounced in PSP-P subtype. Despite some methodological limitations—such as the absence of case–control design and lack of comparisons to other parkinsonian disorders or healthy controls—these findings indicated issues that substantially contributed to a decrease in QoL and could be amenable to symptomatic treatment if recognized early in the disease trajectory.

### 3.1 Scales in parkinsonism (including PSP) for QoL evaluation

Widely used tools for QoL assessment in patients with PSP include the Parkinson’s Disease Questionnaire-39 (PDQ-39) (24), the Progressive Supranuclear Palsy Quality of Life Scale (PSP-QoL) (25), and the EuroQol instrument (EQ-5D) (26, 27). Although the PDQ-39 was primarily designed for PD, it can also be utilized for evaluating atypical parkinsonism conditions, including PSP. However, since it was not tailored for PSP, it fails to address certain prevalent symptoms of the disorder, such as muddled thinking, ocular motor dysfunction, confusion, and apathy (28).

The validation of these instruments for patients with PSP appears to be limited, as they do not encompass the unique aspects of the disease (including several aforementioned symptoms), which may lead to an underestimation of associated health problems (24).

### 3.2 Scales dedicated to PSP for QoL evaluation

The PSP-QoL scale was specifically developed to assess the distinct physical and mental domains experienced by patients with PSP. This scale comprises 45 patient-rated items that evaluate difficulties related to mental and physical tasks within the preceding month. It remains the only tool sufficiently validated for QoL assessment in this disease population (25). Its primary disadvantage is the time-consuming nature of its completion, which restricts its applicability in clinical settings; hence, a more streamlined QoL tool is required. Notably, this scale was developed using a large cohort of patients with PSP, focusing on crucial clinical features and including items with good discriminative power for different disease stages, indicating that the tool has good construct validity and high reliability. As such, it is recommended for use as a patient-reported outcome measure in clinical trials (25). However, the study associated with this tool underrepresented late-stage patients (as they were unable to complete the survey), raising questions about the tool’s applicability to those in advanced stages of the disease. Researchers did not differentiate between probable and possible PSP, as participants were diagnosed outside the study’s centers. Further validation is encouraged in diverse populations. In a study by Patnelyat et al. (29, 30) patients with PSP were evaluated to establish correlations between the PSP Rating Scale and the PSP-QoL. While the study indicated the PSP-QoL as a feasible method of self-assessment and revealed significant associations with the PSP Rating Scale, the authors cautioned that these correlations might differ between patients with Richardson syndrome and those with non-Richardson syndrome. Somewhat addressing the limitations of the PSP-QoL, the PDQ-8—a condensed version of eight representative questions from the PDQ-39 (31)—has gained traction in PD management, including clinical trials (32). However, its effectiveness specifically within the PSP population requires further investigation. In a larger cohort study (*n* = 132), Xin-Yi et al. (33) found that the PDQ-8 correlated well with the PSP-QoL in assessing QoL, indicating strong relevance of the PDQ-8 with each item of PSP-QoL. The authors highlighted that among the short scales used for QoL assessment in patients with parkinsonian disorders, the PDQ-8 was the most comparable to the PSP-QoL. The most significantly affected domains identified were mobility, activities of daily life, cognition, and communication—elements critical not only for the patients themselves but also for their caregivers in terms of recognizing levels of independence.

Further analysis of the impact of several variables (e.g., disease duration, sex, NMSS, ESS, GDS, and MMSE) on the PSP-QoL total score showed that longer disease duration, depression (assessed using the Geriatric Depression Scale [GDS]), and daytime sleepiness (assessed by the ESS) were the most important determinants of QoL. To determine QoL, the established connections between GDS scores, NMSS, PSP Rating Scale (PSPrs), and ESS scores were all confirmed to be crucial for QoL assessment. Similar findings were noted in earlier studies of multiple system atrophy (MSA-P) and PSP (34). The simplicity of the PDQ-8 contributes to a high completion rate, with favorable feedback from patients. Furthermore, detailed comparisons between the two patient groups (PSP-RS vs. PSP non-RS) indicate a worse clinical course of the disease in patients with RS variant, particularly evident in both motor and non-motor symptoms (including deterioration in mood and cognitive function). A strong correlation between the PDQ-39 and PSP-QoL subscales (physical and mental) was also confirmed by Schrag et al. (25), highlighting the relationship between PSPrs and PSP-QoL along with the relevance of non-motor symptoms in patients’ QoL (29). Based on these findings, utilizing both assessment instruments is recommended.

Wiblin et al. emphasized the significance of particular aspects of social life—such as social network strength, family support, and personal disease insight—for the QoL of patients with PSP (17). Among the 19 patient-caregiver pairs studied, reduced QoL was found to be associated with speech difficulties, social life restrictions, and disease stigma. Although their results are based on semistructured interviews rather than formal QoL assessment tools discussed above, they pointed out that further evaluations using standardized scales would allow objective comparisons of patients’ well-being.

The Modified Progressive Supranuclear Palsy Rating Scale (PSPRS) has been devised to quantify and track the severity of major symptoms associated with PSP. In this questionnaire, a physician assesses each of the 28 items categorized into six domains: daily activities (based on patient history), mentation, bulbar symptoms, oculomotor function, limb motor skills, and disturbances in gait/midline (based on examination) (35).

Further evaluations of the tool indicate areas for refinement; for example, some items in the questionnaire reflect critical disease aspects, yet calculating maximum scores for these items does not effectively capture variances in clinical severity. In addition, some of the analyzed items were found to be interrelated or overlapping, indicating they may not need to be evaluated separately. Some items and their scores were also classified as minimally changeable over the course of disease progression, failing to accurately represent changes in a patient’s status (36). For these reasons, the removal of items such as tremors, limb dystonia, and dysphagia has been proposed to improve the consistency of the scale.

A modified version of the PSPRS, referred to as the mPSPRS, was also designed by Grotch et al. (37) by eliminating the following 14 items from the PSPRS that were identified as insensitive or overlapping: Irritability; sleep difficulty, grasping/imitative/utilizing behavior, voluntary left and right saccades, finger tapping, toe tapping, postural kinetics, or rest tremor. This simplification makes patient evaluation less time-consuming while yielding outcomes comparable in metric power to that of the original PSPRS. This modified version was evaluated by experts from the European section of the International Parkinson and Movement Disorder Society. A comparison between the PSPRS and mPSPRS was performed based on data from 86 participants in the TAUROS trial, which examined both baseline and 52-week follow-up data. They reported a strong positive correlation between the mPSPRS and PSPRS total scores. Some differences were observed between patients with RS and non-RS; however, the implications of these differences remain unclear. Given the equivalence, the authors recommend the mPSPRS for disease monitoring due to its demonstrated sensitivity to change and practicality in clinical practice (29).

Researchers highlight the high dropout rate among study participants, attributed to the rapid progression of the disease, which may influence their results. Nevertheless, they assumed that the mPSPRS could effectively depict changes within intervals as short as 52 or even 26 weeks. They also emphasized that the scale does not account for factors beyond emotional incontinence, despite evidence that problems such as bradyphrenia, disorientation, and behavioral changes play a key role in the mental sphere of the disease. Additionally, the study faced challenges in distinguishing between Richardson’s and non-RS forms of PSP, as some recruited patients were diagnosed according to the NINDS-SPSP diagnostic criteria; therefore, the sensitivity to changes in the mPSPRS may vary across these two subpopulations. In rarer forms of PSP phenotypes, a larger sample size would be necessary to detect any changes in mPSPRS scores, and further prospective studies are recommended to confirm the effectiveness of the tool. However, due to its ease of implementation and demonstrated sensitivity to change over time, the authors advocate for the mPSPRS as a valuable resource for routine clinical practice, particularly for assessing treatment effects in forthcoming disease-modifying trials.

The Progressive Supranuclear Palsy Clinical Deficits Scale (PSP-CDS) (38) comprises seven clinical domains: Akinesia-rigidity, Bradyphrenia, Communication, Dysphagia, Eye movements, Finger dexterity, and Gait & balance. Each domain is scored from 0 to 3, indicating no, mild, moderate, or severe deficits. Piot et al. found a good-to-excellent correlation between the total scores from the PSP-CDS and the clinical scales previously discussed for both PSP-RS and variant PSP (vPSP) phenotypes. To date, the PSP-CDS has been evaluated as sensitive, reliable, and easily applicable in both research and clinical contexts.

### 3.3 Other generic scales

In addition to the previously described tools, various generic questionnaires designed for patients PD are also applicable to other conditions. These include the Sickness Impact Profile (SIP), the Medical Outcome Study 36-Item Short Form Health Survey (SF-36), the Life Satisfaction Questionnaire (LSQ), the EuroQol EQ-5D, the WHOQOL-BREF, and the Quality of Well-Being Scale (QWBS) (39–43). All of these tools have been proven to be valid and reliable in assessing PD (41).

The Sickness Impact Profile (SIP) questionnaire was developed to assess 12 areas of functioning at the time of study that are sensitive to disease progression and may not be accurately reflected in other scales (e.g., SF-36, EQ-5D). However, the SIP’s extensive length (comprising 136 items) leads to a completion time of approximately 30 min. The SIP has also shown associations with key constructs relevant to PD, such as the Hoehn and Yahr scale, the Unified Parkinson’s Disease Rating Scale (UPDRS), and the SF-36 (44).

In contrast, the SF-36 is a shorter tool that requires approximately 9 min to complete and evaluates eight aspects of QoL over the 4 weeks preceding the survey. It is particularly effective in predicting disease progression but does possess certain upper and lower thresholds (45). The SF-36 demonstrates good reliability and discriminative validity (41, 46–49) and has been shown to respond partially to changes over time (50) and following intervention (30). One study indicated that it was more responsive than both the PDQ-39 and PDQUALIF (51).

The Life Satisfaction Questionnaire (LSQ) assesses general satisfaction and satisfaction with eight specific life domains, with responses rated on a six-point scale. The EQ-5D scale, with three levels of evaluation, is primarily designed to inform healthcare decision making and detects QoL changes across five health areas, including self-esteem during the study period. While the EQ-5D has a brief completion time of approximately 3 min, it has faced criticism for lacking sensitivity to QoL changes compared to other instruments (43). Nevertheless, some studies have established its correlation with UPDRS and SF-36 scores (52) and its capacity to discriminate between different stages of PD stages (53), as well as to respond to therapeutic interventions (54–56).

The Nottingham Health Profile (NHP) comprises 38 items requiring yes/no responses, covering eight domains. This tool has undergone validation in various populations (57, 58) and has been tested specifically in patients with PD, yielding acceptable validity in this cohort, supported by good internal consistency and a unidimensional factor structure (59). However, the stability of the NHP, as assessed through test–retest procedures, has not yet been evaluated in patients with PD. Unfortunately, floor effects were noticeable in the PDQ-39 results (60). Nevertheless, the NHP is responsive to interventions such as deep brain stimulation (61, 62) and demonstrates changes over time (63, 64).

The QWB system evaluates physical activity, mobility, social activity, and 27 symptoms, categorizing patients into one of 43 functional levels. The assessment is advised to be conducted with the assistance of trained professionals, taking approximately 10–15 min to complete. A specialized version tailored for patients undergoing deep brain stimulation treatment, known as the Questions on Life Satisfaction–Deep Brain Stimulation (QLS-DBS) module, has also been developed. Reports indicate moderate to high convergent validity of the QLS-MD module with the SF-36 and EQ-5D, while moderate convergent validity has been established for the QLSDBS (65).

The Quality-of-Life Questionnaire 15D (15D) comprises 15 questions, each representing 15 distinct dimensions, with five scoring options per question. Although several non-PD populations have indicated good psychometric properties, in the context of PD, the 15D has only undergone partial validation, with one study reporting correlations with the PDQ-39 and UPDRS scores for sections II and III (66).

Among the questionnaires developed exclusively for patients with PD, in addition to the PDQ-39 and PDQL, the PDQUALIF PIMS is a lesser-used questionnaire. This tool contains 32 domain-specific items along with one global item and features seven subscales. It includes many non-motor items, such as autonomic dysfunction, fatigue, sleep disturbances, and sexual function, and has demonstrated sensitivity to changes in clinical trials (67). It is believed that the QoL scales provide greater utility in evaluating the effectiveness of rehabilitation, pharmacological treatments, or neurosurgical interventions in patients compared to other assessment instruments (16).

Table 2 summarizes some strengths and limitations associated with these various assessment tools.

**Table 2:** A simplified overview of scales for QoL assessment.

Scale	Strengths	Limitations
Parkinson’s Disease Questionnaire-39 (PDQ-39) (24, 31)	Assesses difficulties across 8 dimensions of daily living. Telephone interview versions available for remote assessments Good internal consistency	Does not provide a comprehensive overview of certain clinical features of PSP.
Progressive Supranuclear Palsy Quality of Life Scale (PSP-QoL) (25)	Assess items of high clinical significance and importance from the patient’s point of view (19) Highly reliable with good face and construct validity	Responsiveness of the PSP-QoL not tested.
EuroQol instrument (EQ-5D) (26, 27)	Improved sensitivity and reduced ceiling effects. Available in numerous languages and in various modes of administration.	Insensitive to some specific PSP features (19)
Parkinson’s Disease Questionnaire (PDQ-8) (31)	Short-form version, derived from PDQ-39	Non-motor impairment (oculomotor, mental) less evaluated (36)
Modified Progressive Supranuclear Palsy Rating Scale (mPSPRS) (37)	Reflects a wide range of functional disabilities in PSP patients. Good sensitivity to change.	Preferred in clinical trials
Progressive Supranuclear Palsy Clinical Deficits Scale (PSP-CDS) (38)	Uncomplicated formula feasible in clinical practice	-
Sickness Impact Profile (SIP) (44)	Widely covers different functional areas.	Formula requiring longer evaluation time, which may affect its use in clinical settings, particularly among patients with advanced deterioration.
Medical Outcome Study 36-Item Short Form Health Survey (SF-36) (45)	Widely used, well-researched – comparable results from different diseases studied	Largely limited by floor and ceiling effects.
Life Satisfaction Questionnaire (LSQ) (43)	Requires a short completion time in clinical settings.	Not designed for motor disorders Very general findings
Nottingham Health Profile (NHP) (57–59)	Requires a short completion time in clinical settings.	Not designed for motor disorders Very general findings
Quality of Well-Being Scale (QWBS) (65)	Assesses last 3 days of QoL experience.	Not designed for motor disorders Very general findings
Quality-of-Life Questionnaire 15D (15D) (66)	Requires a short completion time in clinical settings.	Not designed for motor disorders Very general findings

PSP, progressive supranuclear palsy; QoL quality of life.

## 4 Limitations

The evaluation of the QoL of patients with PSP is affected by various limitations. Firstly, many of the aforementioned studies were primarily based on clinical examinations, which enabled only probable or possible diagnoses. Neuropathological verification, which provides the possibility of obtaining a definite diagnosis, is rarely performed following the death of patients who have undergone QoL assessments. The relatively rapid progression of PSP and the short life expectancy following diagnosis complicate the characterization of the disease, which is complicated by difficulties in examination of patients with early stage PSP and the limited access to patients with advanced-stage PSP owing to significant cognitive and motor disability. Additionally, owing to the boundaries in the clinical evaluation of patients with advanced-stage PSP, much of the available information stems predominantly from caregivers’ perspectives. Additionally, some studies reviewed herein were conducted prior to the release of the most recent diagnostic criteria established by Hoglinger et al. (4), which may not be as effective in diagnosing patients exhibiting non-Richardson syndrome. PSP being a rare disease makes the interpretation of the statistical outcomes of studies, especially the single-center studies, questionable in the context of the generalization of the results. Despite the notable differences in the disease progression of the two major subtypes—PSP-RS and PSP-P—some studies treat the PSP cohort as a homogeneous group, which may undermine the outcomes of these studies. The COVID-19 pandemic has imposed additional limitations on the examination of patients with significant motor disabilities, such as those seen in PSP. Among other limitations, owing due to limited cooperation with patients with PSP, the assessment of QoL may rely on isolated evaluations and do not allow for trend analyses.

## 5 Summary and perspectives

Based on data from the Adelphi PSP Disease Specific Programme™, a cross-sectional study including 242 patients, their families, and neurologists, Pillas et al. (68), highlighted the substantial burden of disease experienced by patients with PSP, their caregivers, and healthcare systems across all disease phenotypes. In this study, 67–100% of patients across various phenotypes exhibited moderate-to-severe disease severity at the time of enrollment, necessitating care from multiple healthcare professionals and constant care from a minimum of one caregiver. Despite minor statistical limitations—particularly concerning the representation of diverse patient populations and geographical variations—the authors underline the growing involvement of family and healthcare workers in managing disease progression. They also emphasize the pressing need for comprehensive patient management strategies applicable to all phenotypic manifestations of the disease. Adopting an inter-professional approach, healthcare professionals with family support may influence the QoL of patients with PSP (82).

In conclusion, PSP is regarded as an under-recognized and under-researched disease; thus, patients’ multifaceted needs often go unmet. It is recommended that a direct diagnostic journey and systematic follow-up be implemented at specialized movement disorder centers, where individualized education for patients and their caregivers can be provided, which may be potentially affect their QoL. Given the minimal response to symptomatic treatments, rapid disease progression, and short life expectancy, QoL should remain a focal point of attention. As the pathological mechanisms underlying atypical parkinsonism remain unknown, there is a pressing need for further research in this area to explore potential therapeutic interventions and address current patient care requirements. Additionally, more research is warranted regarding QoL, especially within the context of treatments evaluated in clinical trials.
